# A knowledge-based decision support system in bioinformatics: an application to protein complex extraction

**DOI:** 10.1186/1471-2105-14-S1-S5

**Published:** 2013-01-14

**Authors:** Antonino Fiannaca, Massimo La Rosa, Alfonso Urso, Riccardo Rizzo, Salvatore Gaglio

**Affiliations:** 1 ICAR-CNR, National Research Council of Italy, Viale delle Scienze Ed. 11, Palermo, 90128, Italy; 2 DICGIM, University of Palermo, Viale delle Scienze Ed. 6, Palermo, 90128, Italy

## Abstract

**Background:**

We introduce a Knowledge-based Decision Support System (KDSS) in order to face the Protein Complex Extraction issue. Using a Knowledge Base (KB) coding the expertise about the proposed scenario, our KDSS is able to suggest both strategies and tools, according to the features of input dataset. Our system provides a navigable workflow for the current experiment and furthermore it offers support in the configuration and running of every processing component of that workflow. This last feature makes our system a crossover between classical DSS and Workflow Management Systems.

**Results:**

We briefly present the KDSS' architecture and basic concepts used in the design of the knowledge base and the reasoning component. The system is then tested using a subset of Saccharomyces cerevisiae Protein-Protein interaction dataset. We used this subset because it has been well studied in literature by several research groups in the field of complex extraction: in this way we could easily compare the results obtained through our KDSS with theirs. Our system suggests both a preprocessing and a clustering strategy, and for each of them it proposes and eventually runs suited algorithms. Our system's final results are then composed of a workflow of tasks, that can be reused for other experiments, and the specific numerical results for that particular trial.

**Conclusions:**

The proposed approach, using the KDSS' knowledge base, provides a novel workflow that gives the best results with regard to the other workflows produced by the system. This workflow and its numeric results have been compared with other approaches about PPI network analysis found in literature, offering similar results.

## Background

Proteins represent the working molecules of a cell, but to fully understand cell machinery, studying the functions of proteins is not enough. The biological activity of a cell is not defined by the proteins functions *per se *[1], what it is really important is the interactions among proteins.

A group of proteins that interact in order to regulate and support each other for specific biological activities is called a protein complex. Protein complexes are one of the functional modules of the cell: an example of this protein function modules are RNA-polymerase and DNA-polymerase.

The concerted action of different functional modules is responsible of major biological mechanisms of a cellular process such as DNA transcription, translation, cell cycle control, and so on. Since a protein could have several binding sites, each protein can belong to more than one complex and exhibit more than one functionality. The basic element of these modules is the protein-protein interaction (*PPI*). A large amount of PPI data have been identified for different biological species by using high throughput proteomic technologies. Of course experimentalists can take advantage of using different online databases containing a list of PPIs for each species (DIP [2], MIPS [3], etc..), but unfortunately available datasets are still incomplete and contain non-specific (false positive) interactions [4], in fact only a few of interactions have been verified with small scale experiments (*in vitro*) as real interaction with an emerging function.

Usually, in bioinformatics a collection of these interactions is modelled as an undirected graph, the protein-protein interaction network (*PPIN*), where nodes represent proteins and edges represent pairwise interactions: it allows us to exploit graph theory methods and solutions.

The task of exploiting biologically relevant modules in PPINs represents an active research area in bioinformatics, not only for cell understanding, but also for new drugs developing; for example, several authors, as [5], are studying the mechanisms that regulate the evolutionary crossroads of p53 complex, responsible for different aspects of animal life, in developing human cancer cells. Then, identifying protein complexes with emerging function turns into extracting sub-networks with some emerging properties. Because of the importance of isolating functionally coordinated interactions, a lot of models, algorithms and strategies have been introduced to extract interesting PPI subnetwork (soft-clustering, greedy heuristics, probabilistic approaches, etc.), but each of them has proper pros and cons.

A number of clustering-based approaches have been proposed to solve the protein complex prediction problem. A well know algorithm introduced by [6], the Molecular Complex Detection Algorithm (*MCODE*), makes use of local graph properties and it is aimed at finding densely connected regions in protein interaction networks. Another algorithm based on local search is the Restricted Neighbourhood Search Clustering Algorithm (*RNSC*) developed by [7]. This algorithm searches for a low-cost clustering by first composing an initial random clustering, then reducing the clustering cost by a near-optimal strategy. A different strategy is adopted by the Markov Clustering Algorithm (*MCL*) [8], that divides the graph by means of flow simulation. In facts, it separates the graph into different segments, with an iteration of simulated random walks within a graph.

It is possible to increase the reliability of the PPI data by means of preprocessing techniques. Some preprocessing strategies are aimed at eliminating false positive (*FP*) interactions from dataset obtained by online DBs. For example [9] notices that the interactions not part of dense subnetworks, are more likely to be interactions that do not exist. To identify these false positives, authors combined two topological metrics named Cluster Coefficient [10] and Centrality [11]. Also [12] uses the same algorithms, but adopting a different methodology, integrating individual topological measures into a combined measure by computing their geometrical mean. A different approach to improve the quality of PPI datasets is adopted by [13], that attempts to detect those interactions that are missed by large-scale experiments or, in other words, aiming at predicting false negative by means of a topological analysis.

Obviously, the best combination of the proposed techniques depends on the problem and many researchers [12,14-16] have proposed different workflows.

Our approach differs from the previous ones since we face Protein Complex Extraction problem using a Knowledge-based Decision Support System (KDSS). Our KDSS, combining the knowledge extracted from research papers covering a lot of different strategies and methodologies, is able to suggest and run a novel workflow of tasks for the presented issue.

As it will be highlighted in Results and Discussion Section, the suggested workflow, using a test dataset, gives the best results with regard to the other, not suggested, workflows produced by the system and moreover it provides comparable results with respect to some of the common workflows found in literature. From this point of view, our KDSS represents a valid and powerful instrument that can help an experimentalist to face and solve the problem of extracting protein complexes from a PPIN, supporting him in the choice, configuration and running of proper tasks.

## Methods

Knowledge-based DSS is a category of DSS built using an expert system [17]. These systems have their own expertise based on knowledge on many aspects of the problem: the application domain, the definitions of problems within that domain and the necessary skill to solve them [18]. The knowledge of the system is often coded as a set of rules by one or more human experts: this kind of systems are often referred to as rule-based expert systems.

Examples of DSS in Bioinformatics are ProCKSI [19], a system that is able to put together various protein similarity measures in order to obtain the comparison of multiple proteins simultaneously; and INTERPRET [20], a software that offers support in the analysis of Magnetic Resonance Spectroscopy (MRS) data.

Along with the development of Expert and Decision Support Systems, in recent years in bioinformatics a new type of tools, called Workflow Management Systems (WFMS) [21], have begun to spread out. WFMSs provide a simple way to build and run a custom experiment using the most common bioinformatics resources, like online databases, software and algorithms.

The most used and famous WFMS for bioinformatics is Taverna [22]: it is able to automatically integrate tools on databases available both locally and on the web in order to build workflows of complex tasks; to run the workflows and to show results in different formats. The system works by means of a Graphical User Interface (GUI) or a script language. A Taverna plug-in, called Taverna Interaction Service, was introduced in [23]; it extends the functionality of Taverna by defining human interaction within a workflow, once it is running. More in detail, this plug-in acts as a mediation layer between the automated workflow engine and human agents. In facts, it includes a review process, provided by external collaboration partners, invoked by sending emails to target users; they, in turns, can sent back a decision to the workflow produced by Taverna. In this way, users can interact with a piece of data, such as for example an annotation of a genomic region, during the workflow execution.

Other WFMS for bioinformatics are Biowep [24], that allows the user to search and run a predefined set of workflows, already tested, validated and annotated; and BioWMS [25], that is a web-based WFMS built upon an agent-based middle ware architecture.

Cited WFMSs, however, do not have a knowledge base, nor make decision like KDSS; the KDSS we present, on the other hand, offers not only support in the choice of the proper strategy, tool and algorithm, but it helps the user to configure and to run them, step by step. For this reason our system can be seen as an ideal merging point between classical DSS and emerging WFMS. It provides both the tools/services needed to resolve a problem, and also the knowledge necessary to suggest a specific strategy and justify its choice.

### System architecture

First basic ideas of the proposed system can be read in [26,27]: in this Section we will briefly describe its architecture and then we will deepen main concepts at the basis of our KDSS.

**Figure 1:**
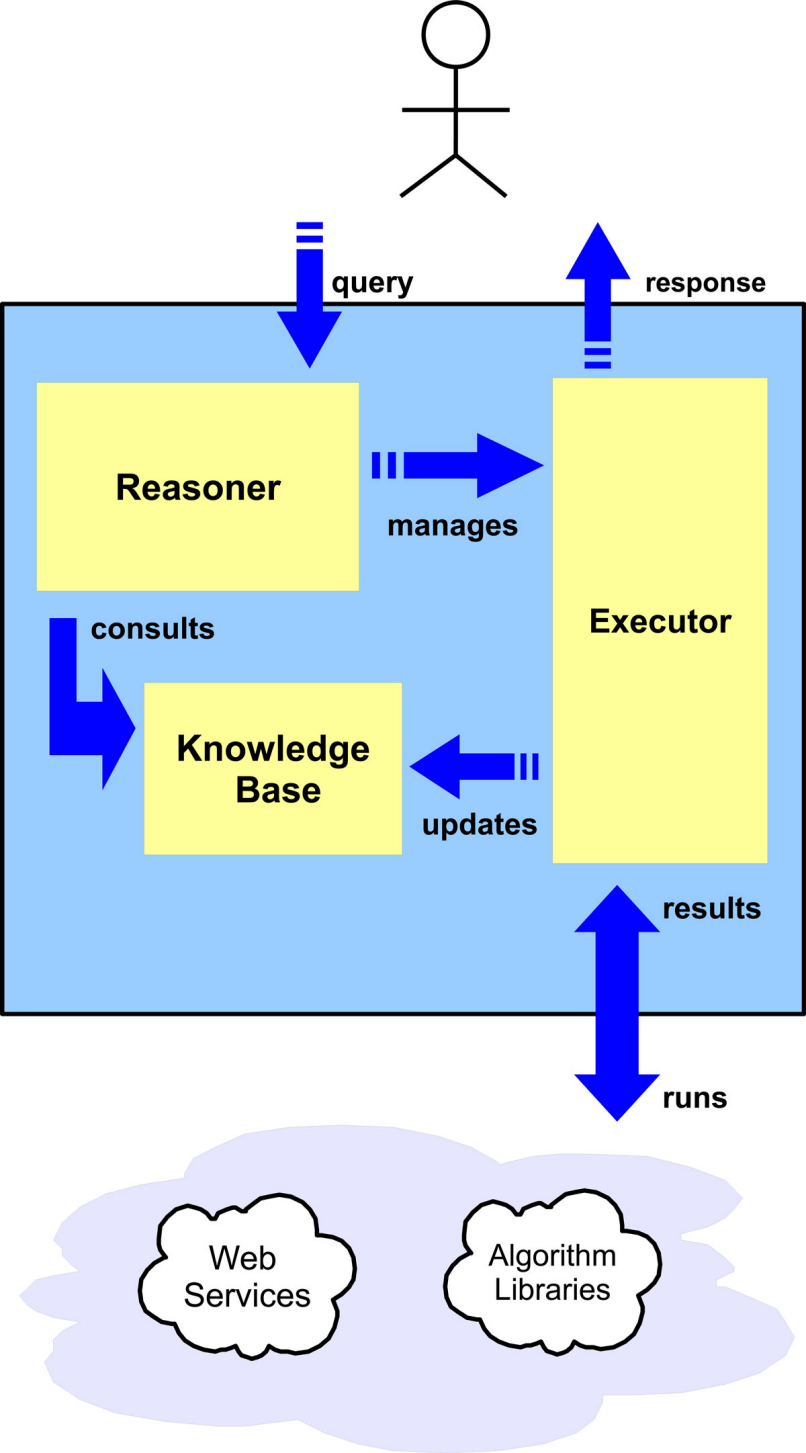
**System architecture**. The system is built upon a rule-based expert system. KB contains the expertise about the application domain in the form of facts and rules. The Reasoner, that is an inference engine, according to the user's requests, input data and available knowledge, decides what are the strategies to follow and the tools to run, and suggest them to the User. The Executor actually runs all the executable processing tools and updates the KB with results of processing, that can be used to make new inferences.

The system core is represented by a rule-based expert system [28]. The three main components of this system are the Knowledge Base (KB), the Reasoner and the Executor: they interact each other as shown in Figure 1.

In the middle part there is the Knowledge Base: it contains all the information, called facts, that encode the expertise of the system about a certain application domain. Facts are given a rigorous and organized structure by means of an ontology of concepts [29].

**Figure 2:**
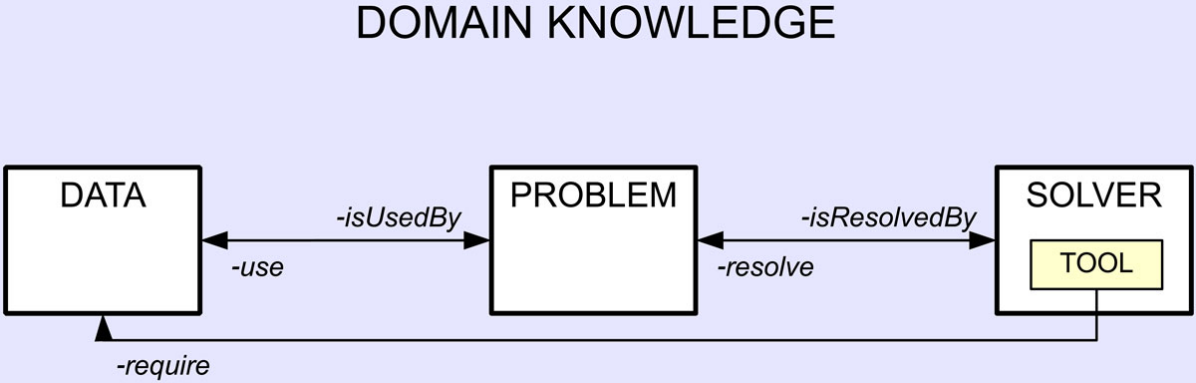
**Data-Problem-Solver ontology for knowledge-based expert systems**. An overview of the Data-Problem-Solver paradigm used for building a complete and exhaustive Knowledge Base.

In order to obtain a well formed Knowledge Base, we adopt a precise and robust organization which, at the same time, is shareable and easily expandable, presented in [30]. In facts, with the introduction of a proper ontology, we can obtain a logical description of a specific problem, share the information among software agents and reuse the specific knowledge domain. In other words, we adopt a paradigm that facilitate the generalization of the application domain and the modularity and the expandability of the represented knowledge. This paradigm, called Data-Problem-Solver (DPS), is able to distinguish and separately model "'what I need"' (Data), "'what to do"' (Problem) and "'how to do"' (Solver), or in other words, I/O data of the problem (Data), the set o tasks (Problem) and the way to solve these tasks (Solver). In this way, we aim at using a very general purpose system based on a KB for rule-based expert systems, that is independent from a specific domain, reusable and expandable. As showed in Figure 2, there is another main element used for solving a specific problem, that is the Tool concept; in facts, an instance of Solver contains information about which tool (or which list of tools) satisfies the purpose (and/or the paradigm) that could solve a specific task. Figure also reports the most important relationship among the tree main branches of the adopted ontology.

Apart from the facts, KB also has a set of rules, in the typical form **IF **<*precondition*>**THEN **<*action*>. The rules, acting on facts, have to be considered as the coding for heuristics, guidelines and strategies adopted by an expert of the domain. Both facts and rules can be provided by one or more experts of the domain or can be extracted from experimental and scientific papers, clinical guidelines and so on.

The Reasoner is an inference engine that uses the facts and rules of the KB in order to make decision: it selects the strategies and the related tools that accomplish the user request according to the actual problem and the input data.

The decision taken by the Reasoner are suggested to the User that can either accept them or select other available strategies and tools. In any case, it is the Executor that actually runs algorithm, tools and services. It can be considered as a sort of interface between the Reasoner and the library of algorithms and processing tools available locally and over the Internet. The Executor also updates the KB with the processing results. New results produce new facts that eventually can trigger other rules.

### Decision making process

Facts and rules of the KB are arranged into a set of decision-making modules, as reported in [26]. In this Section we give a brief explanation of key features of decision-making modules.

The decision-making activity of the system is based on an organization of facts and rules arranged in functional modules called decision making modules. Decision making activity is task oriented. Each module has knowledge and skills, takes care of a specific part of the reasoning process and it is responsible for making decisions about a well defined task. Facts could be shared among different modules, whereas each rule belongs to only a module. Finally, each module can activate a previously defined solver; the same solver could be activated by different modules, by using different rules.

If one module has not the needed knowledge to resolve a part of its task, it can activate another module with the proper skill. This activation mechanism defines a tree structure among decision-making modules, where parent modules manage global and general tasks and children modules are responsible of the decision-making process regarding more specialized tasks.

For example, we can have specialized modules for dealing with preprocessing, visualization, or clustering operations that can be activated by general modules which supervise global task execution. The tree structure of modules can be also represented through a treemap [31]: in a treemap, modules and sub-modules are shown as nested boxes.

### Implementation details

System implementation is based on Java technology; grace to its features, such as platform and location independence, portability, OS independence, Java represents a good support for the proposed work. The system also contains a "Rule-Based System" to manage the knowledge-base; the rule based engine adopted is Jess [32], the Rule Engine for the Java Platform. It supports declarative approach, acting at the decision making level. Jess inference engine uses RETE algorithm [33] as pattern matcher.

The knowledge base have been modeled using one of the largest adopted tool for building ontologies, that is Protege' [34]. Protege' is useful for represent the knowledge used by the proposed architecture, because it implements a methodology for creating ontologies based on declarative knowledge representation systems. Finally, in order to generate and visualize the interactive workflow of the system, we adopt JGraphX Java Swing library [35], that is a product family of libraries providing features aimed at applications that display interactive diagrams and graphs. Among the amount of applications provided by this library, we exploit its functionality related to process diagrams, workflow visualization and flowcharts.

## Results and discussion

The application scenario focuses on the complexes extraction problem, that contains in turns two main sub-problems: the preprocessing and purifying of PPI data and the protein complex clustering.

The rest of this Section is organized as follows: in the next sub-section we introduce the dataset used in the scenario; then we show how the proposed method system integrates aforementioned approaches and how it helps users to face the protein complexes extraction problem. Finally in the last sub-section the analysis of experimental results is discussed.

### Experimental dataset

In our experiments, among different available online databases of PPIs network, we use the Database of Interacting Proteins (*DIP*). The input dataset used in this scenario is a subset of *Saccharomyces cerevisiae *PPI-Network composed by 34 proteins and 90 interactions, as shown in Table 1. This table reports a list of 90 PPIs: for each PPI is shown the uniprotKB ID of the first protein, the uniprotKB ID of the second protein and the *DIP *ID of the interaction between the previous pair of proteins. We chose this very simple dataset because it has been well studied by [36,37] with small scale experiments (*in vitro*) at biological interaction level. *DIP *also provides a subset of PPIs curated manually by experts, that are called core PPIs. A well studied small set of PPI allows us to better describe how the system works, and the choices it takes; obviously we know this dataset is not representative of a whole PPI Network, in facts it represents only a pretext for executing the system and obtaining some results comparable with the other papers in literature [36,38] that use the same dataset.

**Table 1:** Input dataset.

#	Protein_A	Protein_B	PPI_ID
**1**	act1	abp1	DIP-10439E
**2**	app1	abp1	DIP-9959E
**3**	cla4	abp1	DIP-3499E
**4**	sla1	abp1	DIP-2452E
**5**	srv2	abp1	DIP-1139E
**6**	yor284w	abp1	DIP-3500E
**7**	act1	act1	DIP-1145E
**8**	bni1	act1	DIP-1155E
**9**	cof1	act1	DIP-1157E
**10**	las17	act1	DIP-1158E
**11**	pfy1	act1	DIP-1143E
**12**	sla2	act1	DIP-1175E
**13**	act1	aip1	DIP-1140E
**14**	srv2	aip1	DIP-3502E
**15**	hsl7	app1	DIP-3683E
**16**	rvs167	app1	DIP-3907E
**17**	sla2	app1	DIP-3966E
**18**	ysc84	app1	DIP-11282E
**19**	cdc42	bni1	DIP-1154E
**20**	cap2	cap1	DIP-3546E
**21**	gic2	cap1	DIP-3547E
**22**	cla4	cdc42	DIP-2580E
**23**	gic2	cdc42	DIP-2583E
**24**	gic2	cla4	DIP-3639E
**25**	aip1	cof1	DIP-1346E
**26**	app1	cof1	DIP-14613E
**27**	las17	cof1	DIP-1161E
**28**	app1	crn1	DIP-3604E
**29**	cof1	crn1	DIP-11816E
**30**	crn1	crn1	DIP-4127E
**31**	hsl7	hsl7	DIP-9812E
**32**	swe1	hsl7	DIP-7787E
**33**	cap2	las17	DIP-1160E
**34**	las17	las17	DIP-11092E
**35**	rvs167	las17	DIP-3699E
**36**	sla1	las17	DIP-1162E
**37**	sla2	las17	DIP-15438E
**38**	ysc84	las17	DIP-11095E
**39**	bni1	pfy1	DIP-1164E
**40**	bnr1	pfy1	DIP-1166E
**41**	srv2	pfy1	DIP-3762E
**42**	app1	rvs161	DIP-4047E
**43**	las17	rvs161	DIP-4048E
**44**	ybr108w	rvs161	DIP-1781E
**45**	abp1	rvs167	DIP-1138E
**46**	acf2	rvs167	DIP-3900E
**47**	act1	rvs167	DIP-1146E
**48**	rvs161	rvs167	DIP-1780E
**49**	rvs167	rvs167	DIP-3901E
**50**	sla2	rvs167	DIP-10013E
**51**	ybr108w	rvs167	DIP-3902E
**52**	ygr268c	rvs167	DIP-3903E
**53**	yjr083c	rvs167	DIP-3904E
**54**	ypr171w	rvs167	DIP-10016E
**55**	ysc84	rvs167	DIP-10017E
**56**	app1	sla1	DIP-10020E
**57**	rvs167	sla1	DIP-10011E
**58**	srv2	sla1	DIP-10018E
**59**	ygr268c	sla1	DIP-10019E
**60**	yor284w	sla1	DIP-11232E
**61**	ypr171w	sla1	DIP-3964E
**62**	abp1	sla2	DIP-2453E
**63**	cla4	sla2	DIP-3965E
**64**	sla2	sla2	DIP-3144E
**65**	act1	srv2	DIP-1144E
**66**	cof1	srv2	DIP-11822E
**67**	rvs167	srv2	DIP-3029E
**68**	srv2	srv2	DIP-1177E
**69**	trm5	srv2	DIP-4014E
**70**	crn1	svl3	DIP-3603E
**71**	app1	swe1	DIP-4050E
**72**	ygr268c	ygr268c	DIP-2272E
**73**	ysc84	ygr268c	DIP-2243E
**74**	las17	yhr133c	DIP-3700E
**75**	yjr083c	yjr083c	DIP-4186E
**76**	ysc84	yjr083c	DIP-11280E
**77**	rvs167	ynl086w	DIP-3906E
**78**	rvs167	yor284w	DIP-10015E
**79**	sla2	yor284w	DIP-3967E
**80**	yor284w	yor284w	DIP-6160E
**81**	ysc84	yor284w	DIP-11283E
**82**	las17	ypl246c	DIP-3702E
**83**	sla1	ypl246c	DIP-11231E
**84**	cap1	ypr171w	DIP-9981E
**85**	ysc84	ypr171w	DIP-11285E
**86**	abp1	ysc84	DIP-11370E
**87**	acf2	ysc84	DIP-11277E
**88**	sla1	ysc84	DIP-2242E
**89**	sla2	ysc84	DIP-3968E
**90**	ypl246c	ysc84	DIP-11284E

There are 90 PPIs among 34 Proteins for the species *Saccharomyces cerevisiae*. Each row contains two PPIs. For each PPI is shown the first protein uniprotKB ID, the second protein uniprotKB ID and the interaction PID ID between the previous pair of proteins. The complete set of PPIs for this species is available in *Scere20081014.txt *file, provided by PID online database [2].

### System running

**Figure 3:**
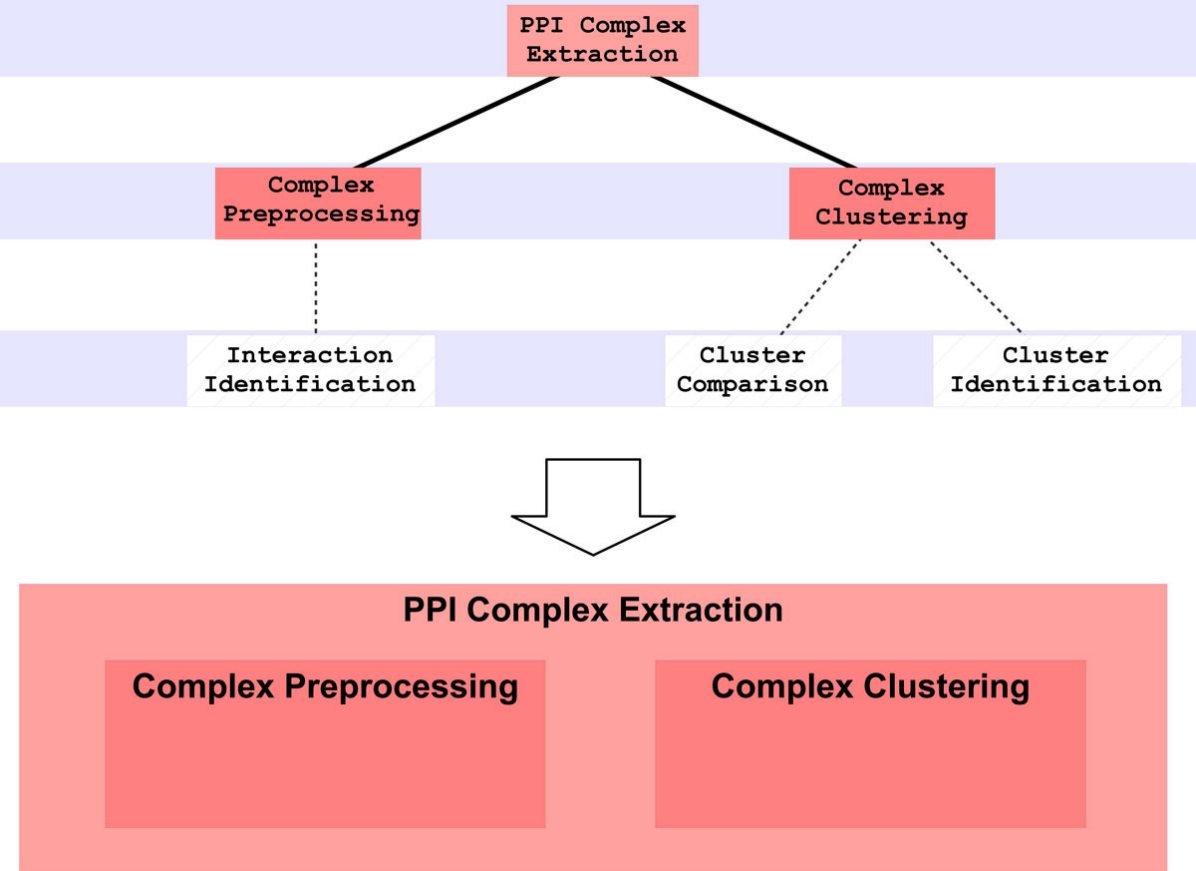
**Decision making modules for Protein Complex Extraction scenario**. The tree structure among modules is projected into a treemap representation. Each parent module is responsible for the activation of children modules. In the treemap, this relation is depicted through a set of nested boxes.

The experiment begins when the user asks the system to extract protein complexes from a PPI-Network and inserts the chosen dataset, the system focuses on decision making modules responsible for the specific problem. More in details, there is the parent module, *PPI Complex Extraction*, that gives directives to two children modules: the first one, *Complex Preprocessing*, contains expertise about PPIN preprocessing, whereas the second one, *Complex Clustering*, has the skill about clustering strategies and tools. This relation is shown in Figure 3, where the decision-making module tree and its treemap representation are presented. Figure 3 also reports other three decision-making modules, namely *Interaction Identification, Cluster Comparison *and *Cluster Identification*, that however will not be activated during the proposed experiment so that they are drawn as dashed boxes. They represent more specialized tasks for improving Complex Preprocessing and Complex Clustering operations. Some guidelines have been extracted from papers cited in Background Section, translated into rules and placed into the appropriate module. The aim of the parent module is to give focus to one of direct children, by means of some activation rules; the system exploits these rules to suggest to the user which strategy could be adopted.

The first operation of the system is to analyze the input data, in order to get the properties and parameters necessary to activate the proper rules. In this simple scenario, we take into account only a few of input features. First of all, the system compares the PPIs of dataset with a list of core interactions, provided by *DIP *for the *Saccharomyces cerevisiae *species. In this case 67 of 90 interactions are reliable, because they are manually curated. Then the system creates the undirected graph, the *PPIN*, and checks if resulting network is scale-free, that is if its degree distribution follows a power law, at least asymptotically. In this scenario the PPIN is not scale-free. Since several authors [39] demonstrate that most networks within the cell approximate a scale-free topology, then some of our PPIs (edges of the network) could be false positives or new PPIs could be not revealed (false negatives) when DIP dataset was created. For this reason, a rule that proposes PPIN preprocessing, in order to change the geometry of the network, is activated.

When the user follows the system advice, according to previous rule, the *PPI Complex Extraction *module gives focus to the child module *Complex Preprocessing*.

According to the analysis phase, the system knows the PPIN contains about 74% of core-interactions. Since it has been estimated that approximately half the interactions obtained from high-throughput proteomic techniques may be false positives [40-42], the rule suggesting to find and delete false positive PPIs is not activated; in fact, cutting edges of PPIN could implicate some core-interactions are deleted and moving core-interactions is lethal for biological networks. For this reason, the rule suggesting to add new PPIs is activated.

When the user agrees to the advice, the system looks for tools implementing this strategy. In this simple scenario, the knowledge-base contains only a tool that can find and add some false negatives (FN) in PPIN: the *Detect Defective Cliques *algorithm, created by [13]. When the user accepts to run the proposed algorithm, then the system informs that this algorithm requires, as input parameter, the number of common interactions between two defective cliques, and suggests to user a considerable value for the experiment.

When the user accepts the proposed value, the system executes the algorithm, that finds a new potential FN interaction between the proteins *act1 *and *sla2*. At this moment, the PPIN is composed by 34 proteins and 91 interactions; the user could either continue the experiment or execute another preprocessing tool (in cascade or restarting the preprocessing phase).

If the user wants to try another solution before continuing the experiment and he does not want to accept the system advices, he could choose to follow the strategy to find and delete false positive PPIs. In this case, the system saves results obtained so far and it proposes to run one of those algorithms that satisfy the selected strategy. The user selects the *Betweenness Centrality *algorithm from among three different tools available into the knowledge-base, because the system indicated this is the algorithm with the lowest computational cost. The result of *Betweenness Centrality *algorithm is a PPIN with 34 proteins, 88 interactions and 65 core-interactions; then the system advices the user to change strategy and/or modify parameters because two core-interactions have been deleted.

**Figure 4:**
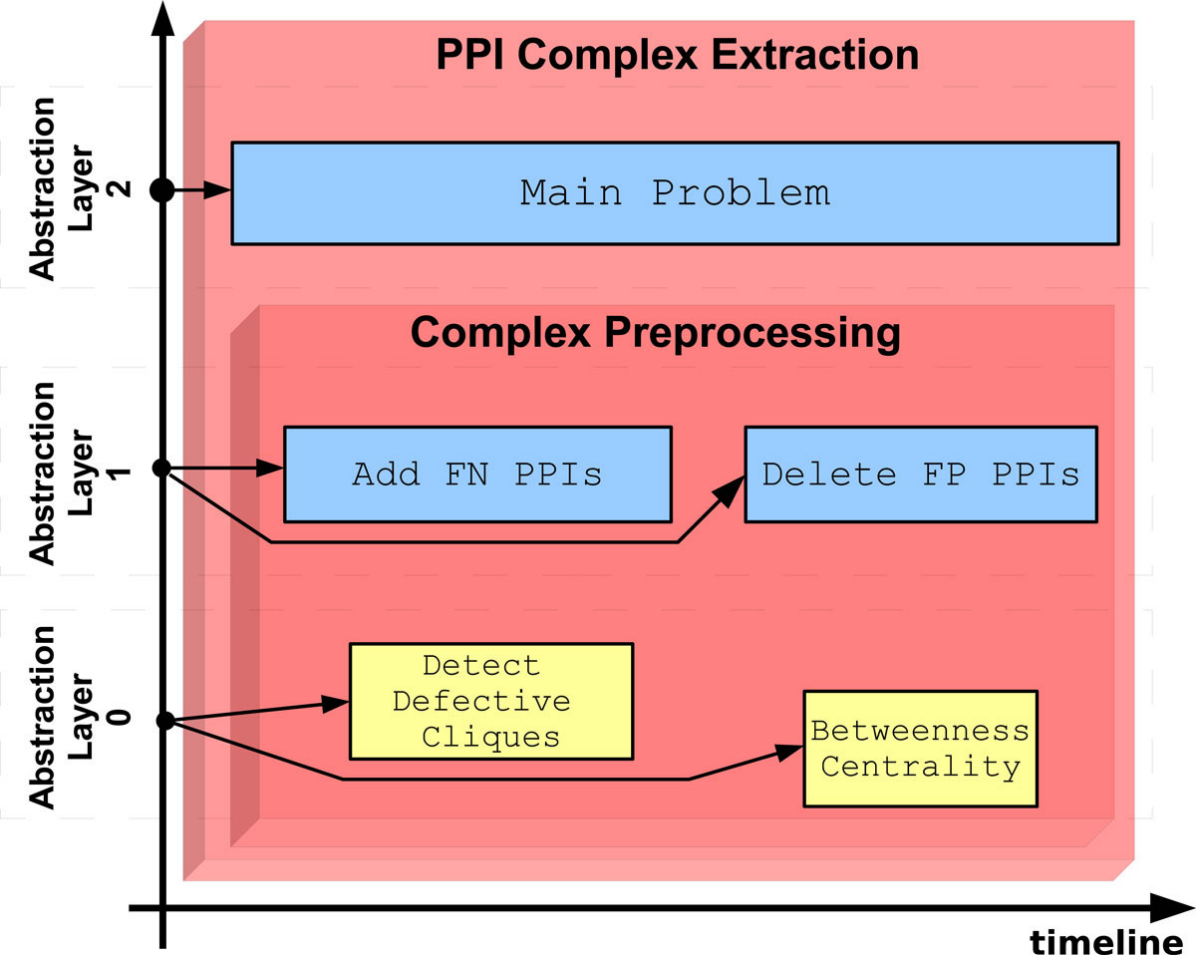
**Workflow of the preprocessing phase**. This figure depict a state of the system during the preprocessing phase, in facts so far two decision making modules are used. The child module, "Complex Preprocessing", reports at "Abstraction Layer 1" the execution of two strategies ("Add FN PPIs" and "Delete FP PPIs") and at lower abstraction layer the executed algorithms (yellow boxes).

Figure 4 shows the workflow our system built so far. The treemap representation of decision-making modules is integrated into the workflow layout. In the figure it is possible to see how *PPI Complex Extraction *module contains all the workflow elements; it supervises the main problem at highest abstraction layer, giving the other directives to *Complex Preprocessing *module. The latter is responsible of some strategies for verifying and purifying the network and have knowledge about tools used for data manipulation. At abstraction layer 1, the child module contains the strategies used in this experiment: in facts the user tried first to add new PPIs and then to delete false positive PPIs; obviously, both these strategies have the same PPIN as input, according to the user choices. All the tools used for processing data are shown at lower abstraction layer and their order in the figure follows the implementation timeline. When the user concludes the preprocessing phase and chooses the appropriate output to continue the experiment, then the *Complex Preprocessing *module ends its activity and gives the focus back to the parent decision module. At this point the *PPI Complex Extraction *module knows the data input has been preprocessed and gives focus to the child *Complex Clustering*. Also the latter module knows the preprocessing phase is done, thus it uses this information for suggesting an appropriate clustering strategy. The authors [12,43] demonstrated *MCODE *is sensitive to noise in the PPIN and the preprocessing phase can increase the algorithm performance. Other authors [14,15] noticed that *MCL *and *RNSC *work almost in the same manner in terms of precision and recall, whether PPIN are noisy or purified. Since *MCL *algorithm is faster than the other algorithms and it work well with dense graphs, the system proposes to use this algorithm based on the flow simulation analysis for clustering. Moreover *MCL *algorithm has been widely used with protein-protein interaction networks belonging to the species *Saccharomyces cerevisiae*, so that the system can suggest standard parameters for this species. When the user accepts the advice and confirms proposed parameters, the system runs the *MCL *algorithm. Now the user can either end the experiment or execute another clustering tool, having as input the PPIN obtained through the preprocessing phase. If the user wants to try another tool, he can consider descriptions, pros and cons that are available for each strategy and algorithm contained into the system.

**Figure 5:**
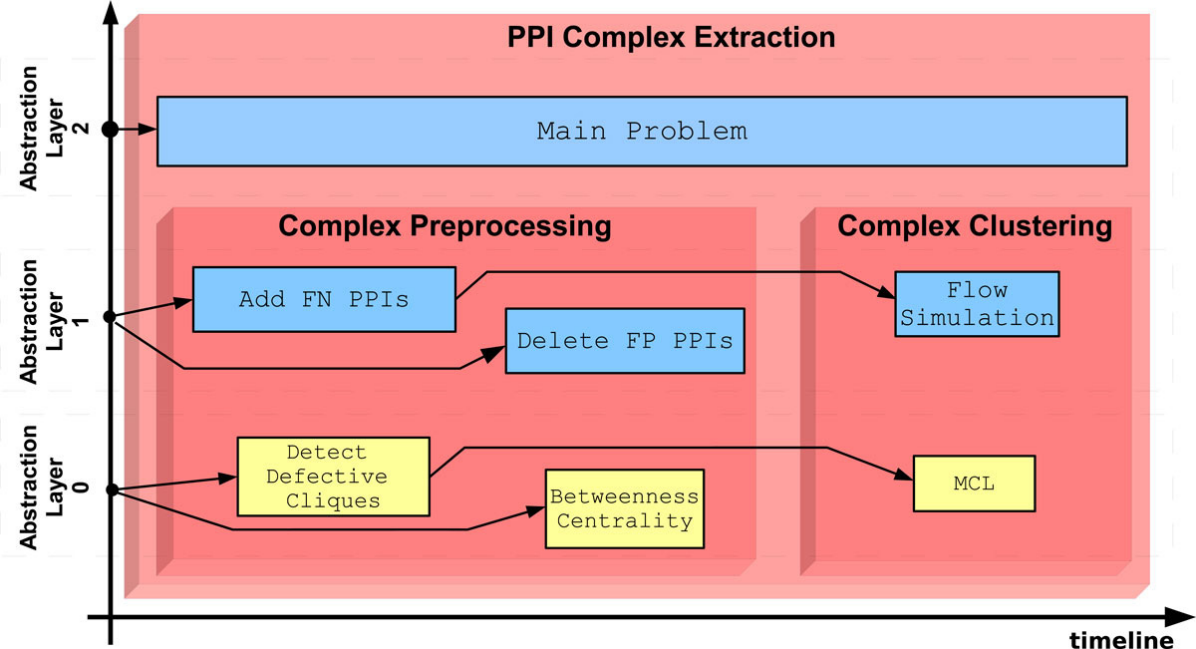
**Workflow of the whole experiment**. The system shows all strategies (blue boxes) and algorithms (yellow boxes) have been used during this scenario. They are arranged in three workflows, one for each abstraction layer. The workflow at "Abstraction Layer 0" reports the complex extraction process at object level.

The whole workflow is shown in Figure 5. At the intermediate abstraction layer, all the strategies within the boundaries of their respective decision modules are depicted, whereas at the lowest abstraction layer there are all the tools implemented in this scenario.

**Figure 6:**
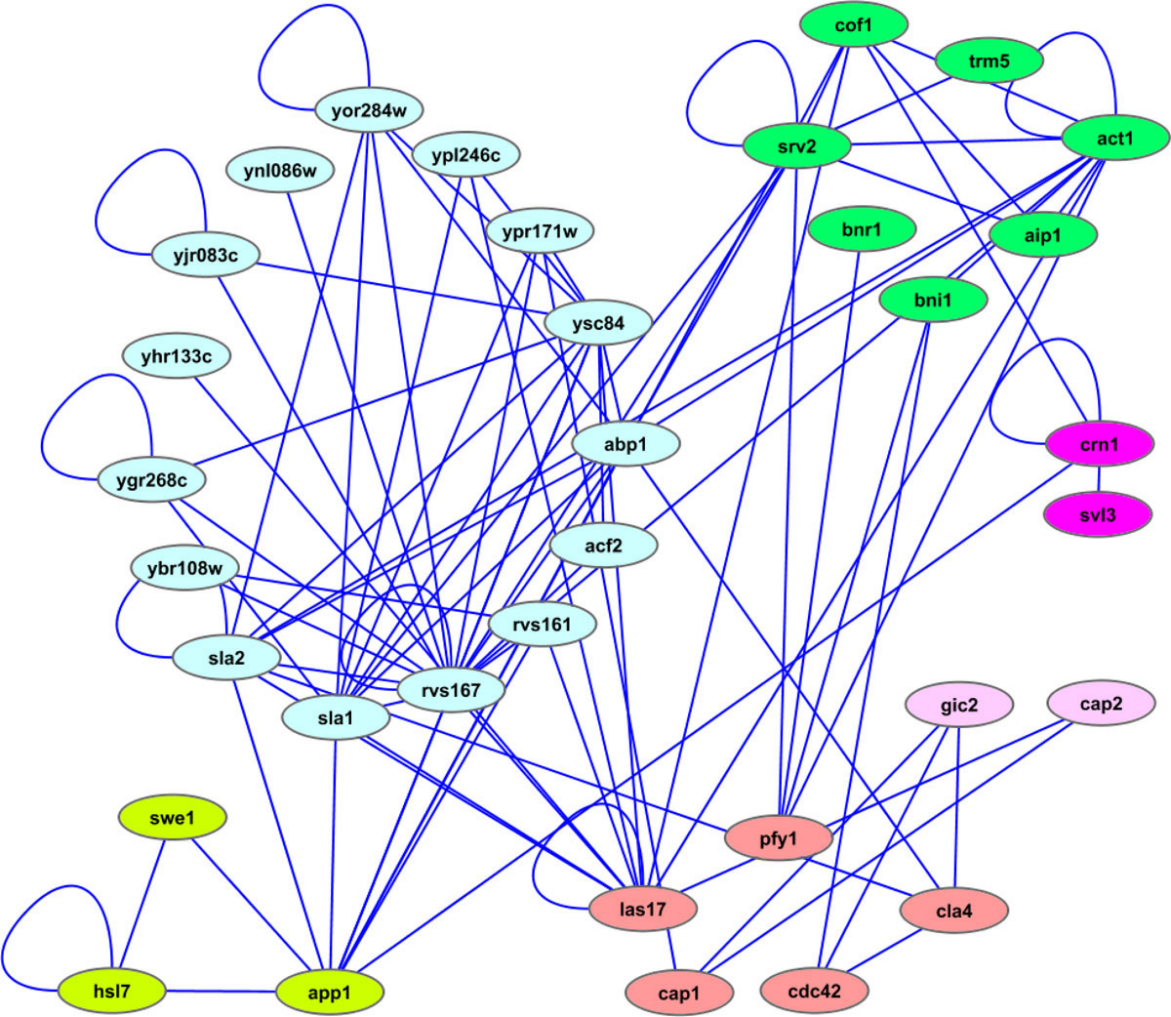
**Clustering visualization with Cytoscape tool**. Cytoscape shows the clustered network arranged in a hierarchical layout. Each complex is depicted in a different colour.

Before concluding the experiment, the system proposes to visualize the output of MCL algorithm with the well know Cytoscape tool [44]. Visualization of clustering results, obtained through Cytoscape, are shown in Figure 6 and reported in Table 2. Finally, the user obtains a solution, that he can further analyze according to its knowledge about the protein complex domain and/or using external evaluation parameters.

**Table 2:** System output.

System Output Detect Defective Cliques + MCL Clustering	
**Cluster**	**Proteins**

1	app1, swe1, hsl7
2	act1, srv2, bnr1, bni1, cof1, trm5, aip1
3	sla2, abp1, yor284w, rvs167, ysc84, sla1, ynl086w, ypl246c, rvs161, acf2, ybr108w, yjr083c, ygr268c, ypr171w, yhr133c
4	cap2, gic2
5	crn1, svl3

The implemented workflow is composed by two algorithms in cascade: Detective Cliques (network preprocessing) and MCL (network clustering). The system running with standard parameters gives five protein complexes as result.

## Discussion

In order to test the result obtained by the system running, the biological significance of each protein complexes is validated by means of the *Gene Ontology Term Finder *web service [45], that returns, for each complex, both the corresponding gene ontology term and the p-value [16]. This statistical measure gives the probability that a group of protein has been clustered by chance: the smaller the p-value, the higher the relationship between a protein complex and the assigned GO term.

Protein complexes obtained by system running are evaluated using two different criteria; in facts, some external and internal evaluation criteria have been analyzed.

The external criterion is based on the comparison among the results obtained through the proposed system and two different approaches proposed by [36,38], that have been previously tested using the best parameter values for the dataset used in this paper. In particular, [38] proposes PINCoC, a co-clustering based approach for protein clustering, whereas [36] introduces UVCLUSTER, an agglomerative hierarchical clustering method: strategies implemented by these two research groups could represent a test-set for the proposed system.

Table 3 reports the comparison among the proposed system and two external approaches, identified with the labels PINCoC and UVCLUSTER: these methods are reported in the first column. The second column shows the proteins belonging to a complex, whereas the next column contains the fraction of proteins that have been identified as responsible of a biological process; finally the last column reports the p-value measure. All the complexes extracted by means of each method are classified in five groups according to related Gene Ontology terms (i.e., *G2/M transition of mitotic cell cycle, Actin Filament Depolymerization, Actin Cytoskeleton Organization, Actin Polymerization or Depolymerization, Rho Protein Signal Trasduction*). Table 3 demonstrates that the system suggests a workflow of operations and the related parameters that are able to reach considerable results with respect to the other approaches. More in details, our system works better than the other methods for the second and the third complex, in facts the proposed KDSS reaches the best values of p-value respectively with 4.92e-10 and 4.65e-08, whereas is not able to find any cluster belonging to the GO term *Rho Protein Signal Trasduction*.

**Table 3:** Comparison among the proposed approach and some of the other approaches.

Methods	Protein complexes	Protein fraction	p-Value
***G2/M transition of mitotic cell cycle***			

Proposed System	app1, swe1, hsl7	2/3	2.17e-03
PINCoC	swe1, hsl7	2/2	6.90e-04
UVCluster	app1, swe1, hsl7	2/3	2.17e-03

***Actin Filament Depolymerization***			

Proposed System	act1, srv2, bnr1, bni1, cof1, trm5, aip1	4/7	4.92e-10
PINCoC	bnr1, bni1, pfy1, act1, srv2, aip1, trm5	5/7	1.52e-07
UVCluster	act1, srv2, aip1, trm5, cof1	4/5	7.30e-04

***Actin Cytoskeleton Organization***			

Proposed System	sla2, abp1, yor284w, rvs167, ysc84, sla1, ynl086w, ypl246c, rvs161, acf2, ybr108w, yjr083c, ygr268c, ypr171w, yhr133c	7/15	4.65e-08
PINCoC	sla2, abp1, yor284w, rvs167, ysc84, app1, rvs161, ynl086w, yjr083c, acf2	6/10	6.72e-08
UVCluster	sla2, abp1, yor284w, rvs167, ysc84, sla1, ygr268c	4/7	5.93e-05

***Actin Polymerization or Depolymerization***			

Proposed System	crn1, svl3	0/2	> 0.01
PINCoC	crn1, svl3, las17, yhr133c, cof1	3/5	9.07e-06
UVCluster	crn1, svl3	0/2	> 0.01

***Rho Protein Signal Trasduction***			

Proposed System	----	---	---
PINCoC	cdc42, cla4, gic2	3/3	1.76e-06
UVCluster	cdc42, cla4, gic2	3/3	1.76e-06

The table reports a comparison among the proposed approach and two different approaches, called respectively PINCoC and UVCluster, that have been previously tested with this database. Proposed system outperforms result of the other tools with respect to the complexes *Actin Filament Depolymerization *and *Actin Cytoskeleton Organization*.

With regard to the internal criteria, two different tests have been executed, aiming at demonstrating the workflow suggested by the system is the best than the other workflows the system could build, according to its knowledge. In particular, the first test reports a comparison among all the algorithms contained in the knowledge base of our KDSS for the complex clustering problem: MCL, RNSC and MCODE. It is worth remembering that the proposed scenario contains only three of the most common and high-performance algorithms for protein complex extraction, because they represent a simple set of tools able to demonstrate how good the system works. Results of the first test are shown in Table 4; the structure of the table is the same as the previous table. It is possible to see as the tool suggested by the proposed system reaches the smallest p-value for all the functional groups but the last cluster, where the proposed tool with standard parameters does not exhibit any result. This test is enough for demonstrating the system suggests the appropriate algorithm to the best of its knowledge.

**Table 4:** Comparison among some different clustering strategies for protein complex problem.

Techniques	Protein Complexes	Protein Fraction	P-Value
G2/M TRANSITION OF MITOTIC CELL CYCLE			

MCL **(suggested)**	app1, swe1, hsl7	2/3	2.17e-03
RNSC	app1, swe1, hsl7	2/3	2.17e-03
MCODE	--	2/3	2.17e-03

ACTIN FILAMENT DEPOLYMERIZATION			

MCL **(suggested)**	act1, srv2, bnr1, bni1, cof1, trm5, aip1	4/7	4.92e-10
RNSC	act1, srv2, aip1, cof1	4/4	2.94e-05
MCODE	--	--	5.25e-03

ACTIN CYTOSKELETON ORGANIZATION			

MCL **(suggested)**	sla2, abp1, yor284w, rvs167, ysc84, sla1, ynl086w, ypl246c, rvs161, acf2, ybr108w, yjr083c, ygr268c, ypr171w, yhr133c	7/15	4.65e-08
RNSC	sla2, yor284w, rvs167, ysc84, sla1, ygr268c, abp1	4/7	5.93e-05
MCODE	abp1, app1, rvs167, act1, yor284w, ysc84	4/6	1.93e-05

ACTIN POLYMERIZATION OR DEPOLYMERIZATION			

MCL **(suggested)**	crn1, svl3	0/2	> 0.01
RNSC	crn1, svl3	0/2	> 0.01
MCODE	--	0/2	> 0.01

RHO PROTEIN SIGNAL TRASDUCTION			

MCL **(suggested)**	--	--	--
RNSC	cla4, bni1, cdc42, gic2	3/4	1.04e-05
MCODE	cla4, cdc42, gic2	3/3	1.76e-06

Table reports a comparison among three clustering techniques contained into the knowledge base of the system: MCL, RNSC and MCODE. The suggested tool allows the system to reach the smallest p-values for all the complexes, but the *Rho Protein Signal Trasduction *cluster.

The second test aims at investigating about the preprocessing phase suggested by the system. In facts the system, according to its knowledge, can deal with the complex extraction problem using a preprocessing of the input PPI-Network, by means of two strategies: finding the false negative PPI (adding edges to the network) or the false positive PPI (removing edges from the network). Since the MCL algorithm proposed by the system is not sensitive to all three of the alternative ways related to the network preprocessing, we test the result of the preprocessing phase over the MCODE tool, because a comparison of clustering algorithms for protein-protein interaction networks showed that MCODE is sensitive to noise in the network [43]. For this reason, MCODE is a suitable candidate for evaluating the effect of network preprocessing. In this scenario, the system proposes the first strategy and suggests to use the "detect defective cliques" tool. Table 5 shows results of this last test. The first column contains the available preprocessing techniques; the second column reports the effect of the strategy on the network; next column reports the set of proteins for each clusters; the fourth column reports the fraction of proteins that have been identified as responsible of a biological process and the last column reports the p-value measure. Table 5 shows the suggested algorithm reaches a smaller p-value (*1.93e-05*) in the complex related to the *Actin Cytoskeleton Organization *GO term, therefore the system proposes once again the most appropriate algorithm to the best of its knowledge.

**Table 5:** Comparison among three different preprocessing techniques when MCODE tool is selected.

Preprocessing Methods	PPI-Network Modification	Protein Complexes	Protein Fraction	P-Value
**Actin Cytoskeleton Organization**				

Detect Defective Cliques **(Suggested) **	Add 1 PPI	abp1, app1, rvs167, act1, ysc84	4/6	1.93e-05
No Preprocessing	---	abp1, app1, rvs167, act1, yor284w	3/5	8.10e-04
Betweenness Centrality	Remove 3 PPI (2 core PPI)	abp1, rvs167, ysc84, yor284w	2/4	7.1e-05

**Bipolar Cellular Bud Site Selection**				

Detect Defective Cliques **(Suggested) **	Add 1 PPI	sla2, las17	2/2	5.30e-04
No Preprocessing	---	sla2, las17	2/2	5.30e-04
Betweenness Centrality	Remove 3 PPI (2 core PPI)	---	---	---

**Rho Protein Signal Trasduction**				

Detect Defective Cliques **(Suggested) **	Add 1 PPI	cla4, gic2, cdc42	3/3	1.76e-06
No Preprocessing	---	cla4, gic2, cdc42	3/3	1.76e-06
Betweenness Centrality	Remove 3 PPI (2 core PPI)	cla4, gic2, cdc42	3/3	1.76e-06

Table reports a comparison among some of the preprocessing tools contained into the knowledge base of the system.The suggested tool allows the system to reach the smallest p-values for all the complexes.

## Conclusions

In this paper, we presented a novel approach for the extraction of the protein complexes based on KDSS. The system interacts with the user using its expertise about PPINs. The system suggests to the user what are the strategies and algorithms suitable for the problem and, moreover, helps him providing the description, pros and cons of each available technique. Finally the system also runs the selected tools, suggesting to the user what are the most common parameters for the specific situation and, during the experiment it builds a workflow of executed operations, enabling the chance of backtracking for exploring alternative paths. The presented results show that the workflow suggested by the system gives the best results with regards to the other workflows produced by the system itself and, furthermore, that workflow offers similar results when compared to other PPI extraction methodologies found in literature.

### Future work

In the near future, we will give research community free access to our system, thanks to the migration towards a web service. Moreover we are working in order to enrich the Knowledge Base with skill regarding the proposed scenario and, at the same time, we will use our system architecture for facing other bioinformatics issues. Finally we will give support to the developer community in order to provide a simple editor so that it will be possible to insert into the system further knowledge and expertise.

## Competing interests

The authors declare that they have no competing interests.

## Authors' contributions

AF: software design, implementation, writing, assessment, discussions. MLR: software design, implementation, writing, assessment, discussions. RR: software design, discussions, writing. AU: software design, discussions, writing, funding. SG: project conception, software design, discussions. All authors read and approved the final manuscript.

## Declarations

The publication costs for this article were funded by the CNR Interomics Flagship Project "-Development of an integrated platform for the application of "omic" sciences to biomarker definition and theranostic, predictive and diagnostic profiles".

This article has been published as part of *BMC Bioinformatics *Volume 14 Supplement 1, 2013: Computational Intelligence in Bioinformatics and Biostatistics: new trends from the CIBB conference series. The full contents of the supplement are available online at http://www.biomedcentral.com/bmcbioinformatics/supplements/14/S1.
